# Role of irradiated and un-irradiated alginate as edible coating in physicochemical and nutritional quality of cherry tomato

**DOI:** 10.1186/s12870-024-05893-w

**Published:** 2024-12-26

**Authors:** Amina A. Aly, Rabab W. Maraei

**Affiliations:** Natural Products Research Department, National Center for Radiation Research and Technology, Egyptian Atomic Energy Authority, Cairo, Egypt

**Keywords:** Cherry tomato, Sodium alginate, Irradiation, Storage condition, Physico-chemical, Nutritional quality

## Abstract

**Background:**

Fresh vegetables are commodities that have a high tendency to deteriorate after harvest, causing significant losses in economic and environmental costs associated with plant food loss. Therefore, this study was carried out to evaluate the effects of both un-irradiated (UISA) and irradiated sodium alginate (ISA) as an edible coating for preserving cherry tomato fruits under storage conditions. The FTIR, XRD, TGA, SEM, and TEM were used to characterize the UISA and ISA (25, 50, 75, and 100 kGy), which demonstrated that the alginate polymer was degraded and low molecular-weight polysaccharides were formed as a result of irradiation, particularly with the 100 kGy dose level. Sodium alginate irradiated at 100 kGy was used for the coating process, and the physico-chemical and nutritional quality of cherry tomatoes were analyzed.

**Results:**

The results demonstrated that UISA and ISA treatments delayed changes in most of the ripening characteristics; weight and acidity losses, decay, and softening. The weight loss of the control was observed to be greater at the two keeping temperatures (4 and 25 °C) comparison with tomatoes coated with UISA. The ISA coatings gave the least weight loss at the two keeping temperatures (4 and 25 °C) (5.46 and 14.72%), respectively compared to the control (8.77 and 18.93%), respectively at the end of the storage period. In terms of antioxidant properties, significant results were obtained with the use of the alginate coating, specially irradiated sodium alginate. Over time, the majority of water-soluble vitamins in cherry tomatoes decreased, especially vitamin C, and the alginate-coated tomatoes showed the least decrease in vitamin C content, especially ISA.

**Conclusions:**

The current findings suggest that ISA treatment efficiently extends the storage period of tomato fruits and maintains their quality through preservation and offers promising potential for successful commercialization of this eco-friendly eatable coating for fruit and vegetable growers and industries.

## Background

Postharvest food loss has become one of the major problems in food security in developing countries. Although scientific and technological advancements have been made in post-harvest preservation technology, postharvest loss remains a common problem all over the world, which is primarily due to the complexity and high cost of the existing methods and materials [1]. About 1.3 billion tons of agricultural production are lost or wasted each year due to postharvest losses, which is equivalent to one-third of total agricultural production. Reducing waste and loss could therefore be a long-term option to increase food supply and improve farmers’ livelihoods without placing additional pressure on natural resources [2]. The consumption of fresh fruits has sharply enhanced in the past century, which triggered a commercial requirement for better logistical and warehousing elements to preserve the quality of fresh products (such as flavor, color, nutritional aspects, shelf life, and processing characteristics), meanwhile, controlling the spread of postharvest diseases of fresh products during their storage period [3]. Egypt is the fifth largest producer of tomatoes in the world, and tomatoes are a very important crop in Egypt in terms of consumption and income for smallholder farmers [4]. Cherry tomato is among the most widely consumed types of tomatoes, which are aromatic and red-colored, with a hard texture and small size [5]. They are also well-known for their flavor and dietary value throughout the world [6]. However, it is a climacteric fruit and highly perishable [7]. According to Aly et al. [8], the main factors that affect its quality include ethylene production, environmental conditions, and deterioration caused by fungi. The rate at which tomatoes ripen is controlled by temperature and humidity; eventually, the fruits become inedible due to softening of the flesh [9]. The need for natural alternatives that extend fruit shelf life has increased over the past few decades as a result of the increasing demand for fresh fruits and vegetables free of artificial stabilizers [10]. A number of techniques have been used over time to maintain the quality and safety of cherry tomatoes, which are climacteric fruit with a short shelf life [3]. Various approaches, including resistance hybrids, fungicides, and agricultural requirements are commonly employed as commercial strategies to inhibit postharvest diseases. However, the use of fungicides intensively may help pathogens to be resistant, as well as fungicides released to the environment [11]; nonetheless, it may also release risks to human health and the environment [12]. According to Wu et al. [12], the application of this technique is becoming more limited to address consumer concerns and the significance of human health. Other techniques, such as ethanol vapor treatments and controlled atmosphere, have increased tomato shelf life [13]. Among several postharvest technologies available, edible coating has been shown as an effective novel postharvest technology that has varied applications in various food products [14], and it is commonly used as a low-cost, available and convenient method for preserving many products [15]. The coatings used to prolong the postharvest storage of different crops are based on polysaccharides, proteins, and lipids. Edible coatings based on polysaccharides, like chitosan and alginate, have grown and become more popular lately. They act as barriers against H_2_O vapor, microorganisms, and atmospheric gases, helping to reduce respiration and oxidation reaction rates [16]. They also act as barricades during handling, processing, and storage and do not exclusively delay food deterioration but also enhance the quality of the product. Furthermore, they are also safe owing to the integration of antimicrobial compounds or the coating’s natural biocide activity [3]. Moreover, edible coatings are considered eco-friendly since they replace plastic packaging and reduce food waste by increasing the shelf-life of food products [17]. Alginate is a polysaccharide of marine origin extracted from brown algae, and it is categorized as a generally regarded as safe compound by the US Food and Drug Administration (FDA), recorded as an approved food additive by the European Commission (EC), and utilized as an emulsifier, stabilizer, thickener, and gelling agent in the food industry [18]. Alginate has good biocompatibility, edibility, and outstanding film-forming properties, so it is widely used in various edible films. However, in the presence of multivalent cations, such as calcium ions, alginate is prone to gelation and becomes insoluble due to the formation of cross-linked structures [19]. According to Nair et al. [20], alginate coatings were able to preserve a variety of fruits and vegetables, such as cherry tomatoes. A study conducted by Salama and Aziz [21] succeeded in extending the storage period of tomato when coated by alginate supplemented with titanium oxide nanoparticles and then exposed to UV light. There are no studies on the use of irradiated sodium alginate as an edible coating for increasing the shelf life and maintaining the quality attributes of postharvest fruits and vegetables. Hence, in the current work study the effect of un-irradiated and irradiated sodium alginate coatings on cherry tomatoes’ shelf life and physicochemical as well as nutritional value during storage period at 4 and 25 °C.

## Results and discussion

### Characterizations of sodium alginate (SA)

It is well known that polysaccharides in dry form or in solution degrade when exposed to ionizing radiation [22]. Irradiation processing offers a clean one step method for the formation of low molecular weight polysaccharides and it can induce degradation of natural polymers like alginate [23].

### Fourier transform infrared (FTIR) spectroscopy and X-Ray diffraction (XRD) analysis

The fourier transform infrared (FTIR) method is a type of spectroscopy that can detect changes in the total composition of biomolecules by determining changes in functional groups [24].

**Fig. 1:**
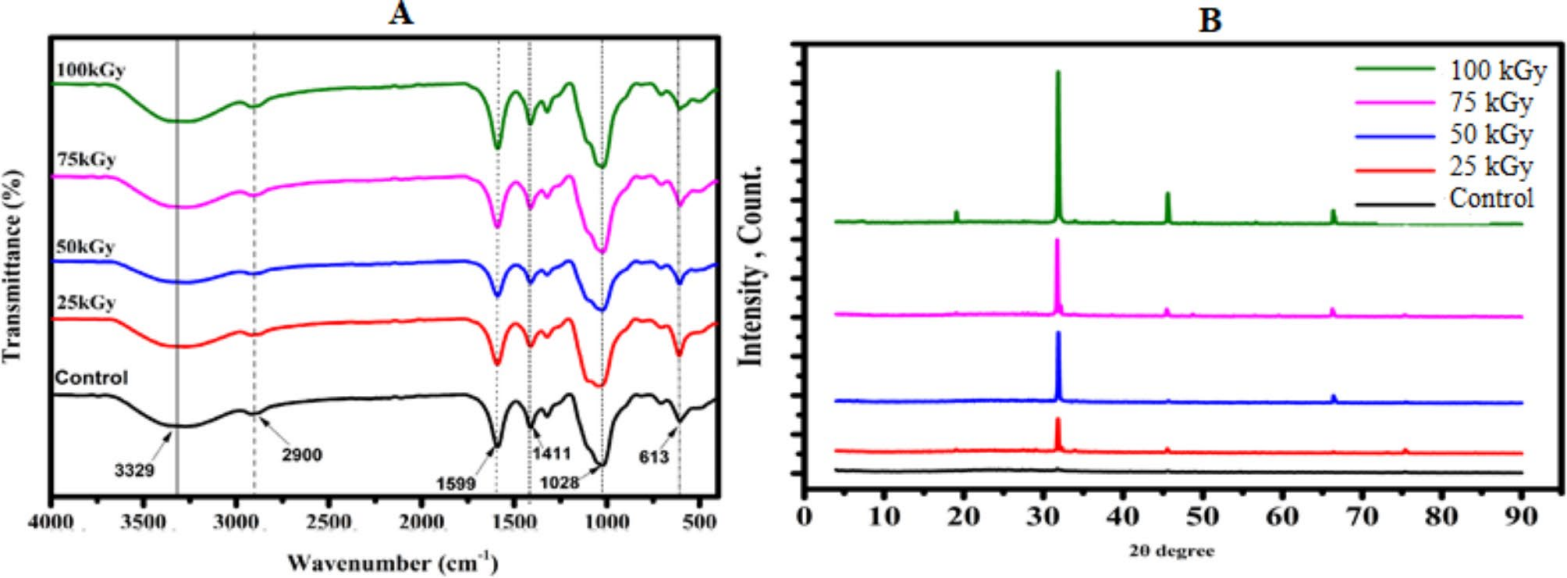
Fourier transform infrared (FTIR) spectrum (**A**), X-Ray diffraction (XRD) spectrum (**B**), of UISA and ISA (100 KGy)

Results indicated by Fig. 1A showed that FTIR spectrum of sodium alginate that has been exposed to varying doses of gamma irradiation (25, 50, 75, and 100 kGy). The un-irradiated SA showed the polysaccharides structure characteristic absorption bands at 1028 cm^− 1^ for C–O stretching and at 3329 cm^− 1^ for OH [25]. At 1599 and 1411 cm^− 1^, respectively, asymmetric and symmetric stretching of carboxylate vibrations was observed [26]. Alginate’s peak at 1599 cm^− 1^ was taken as the reference peaks due to the fact that carboxyl groups do not change after degradation. The spectra of ISA exhibited most of the characteristic adsorption peaks of native alginate but with some differences. The spectra indicated the formation of new C‚ O and OH groups suggesting that ionizing radiation treatment lead to the scission of glycosidic bonds with the change of the structure of reducing end residue. This is manifested as an increase in the ratio of OH group peak and broad C‚ O peak to the reference peaks. Simultaneous decreasing of the peak ratio of C–O–C group to the reference should be perceived. These peaks form as a result of gamma ray-induced glycosidic bond cleavage and hydrogen abstraction, followed by ring opening [25, 27]. The findings of the recent investigation coincide with earlier investigations [25, 28]. In the same concern, Singh et al. [29] reported that, the increased peak intensity in gamma irradiated sodium alginate (ISA), implies the formation of new C = O and –OH groups as a result of glycosidic bond scission with a change in the structure of the reducing-end residue.

Regarding XRD analysis, it made to illustrate the structural changes on SA treated with gamma rays. Patterns in Fig. 1B showed that the X-ray diffraction of un-irradiated SA and that exposed to irradiation doses of 25, 50, 75 and 100 kGy. Un-treated and treated alginate exhibited a characteristic peak at 2θ = 31.8 but there is a reduction in its value and the intensity of this peak with increasing irradiation dose. Also, the full width at half maximum (FWHM) decreased by increasing irradiation dose level from 0.2097º for 25 kGy to 0.1629º at 100 kGy.

This means that irradiation process caused degradation and destruction in SA structure. The degradation was probably due to the direct effect of radiation and the indirect effect due to oxidation process. Irradiation of polysaccharides leads to breakdown of the ordered system of intermolecular hydrogen bonds. Consequently, the rigidity of chains is influenced by intra-molecular hydrogen bonding and the degree of crystallinity of the material decreases. It was assumed that the degradation first took place preferentially in the amorphous region and then proceeded very moderately from the edge to the inside of the crystalline at higher doses (above 100 kGy) [25]. The analysis of X-ray diffraction patterns showed amorphization of solid alginates during gamma irradiation treatment [30]. The recent findings are in line with those of Aly et al. [23], who observed that SA exposed to radiation resulted in the depolymerization of SA into small molecular-weight oligosaccharides. Furthermore, Singh et al. [29] established that, the decrease in the intensities of these peaks after irradiation indicated that irradiation caused the degradation of chitosan and sodium alginate through the breakdown of intermolecular hydrogen bonds, resulting in a decrease in their crystallinity.

### Thermal gravimetric analysis (TGA)

**Fig. 2:**
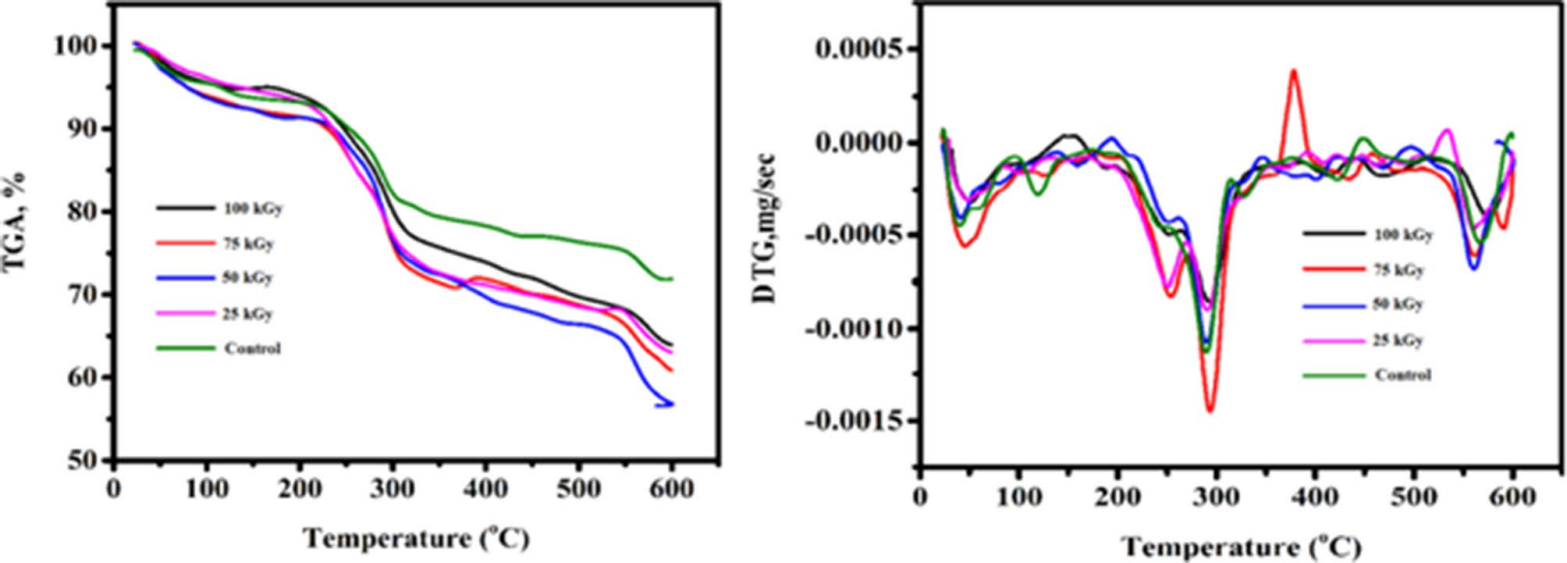
Thermogravimetric analysis (TGA) spectra of un-irradiated (UISA) and irradiated (100 KGy) sodium alginate (ISA)

The weight loss percentage and TGA curves of both un-irradiated and gamma irradiated SA at varying doses is displayed in Fig. 2 and Table 1. It served as a tool for improving comprehension of thermal stability. The outcome shows that there were three phases of weight loss. The first one starts below 50 ºC, it assigned to loss of water molecules that interact with OH and –COO– polar groups in alginate chain by hydrogen bonding, since a considerable amount of water is released at temperatures below 50 ºC for UISA and 40 ºC for 100 kGy. The second stage was the melting point which was 293 ºC for un-irradiated SA and 289 ºC for 100 kGy. The third stage was the oxidation point which was 575 ºC for un-irradiated SA and 567 ºC for irradiated SA. From Table 1, it was observed that sodium alginate irradiated with 100 kGy gave the minimum weight reminder (28.13%) compared to the control (36.06%). There is a noticeable change between the thermal stability of irradiated SA with un-irradiated one [25]. Moreover, Aly et al. [23] found that, irradiated SA with the dose of 100 kGy gave the small molecular weight oligosaccharides. Additionally, it was found that there is a significant change in the thermal stability of sodium alginate irradiated at different doses compared with native one whereas no significant difference occurred among each other [30]. The decrease in crystallite size was due to change in full width at half-maximum (FWHM) value as dose increases. This is mainly due to intrinsic defects in the polymeric crystals which are dominant over gamma irradiation [31].

**Table 1:** Reminder weight of un-irradiated and irradiated sodium alginate after TGA

Dose (kGy)	Weight % at 600 ^o^C	Reminder weight %
Control	63.94	36.06
25	60.88	39.12
50	56.60	43.40
75	63.06	36.94
100	71.87	28.13

From the above, it is clear that sodium alginate irradiated with 100 kGy gave the best properties and also gave the lowest reminder weight. Therefore, a SEM and TEM of this dose was conducted compared to un-irradiated alginate to clarify this.

### Scanning electron microscopy (SEM) and transmission electron microscopy (TEM)

**Fig. 3:**
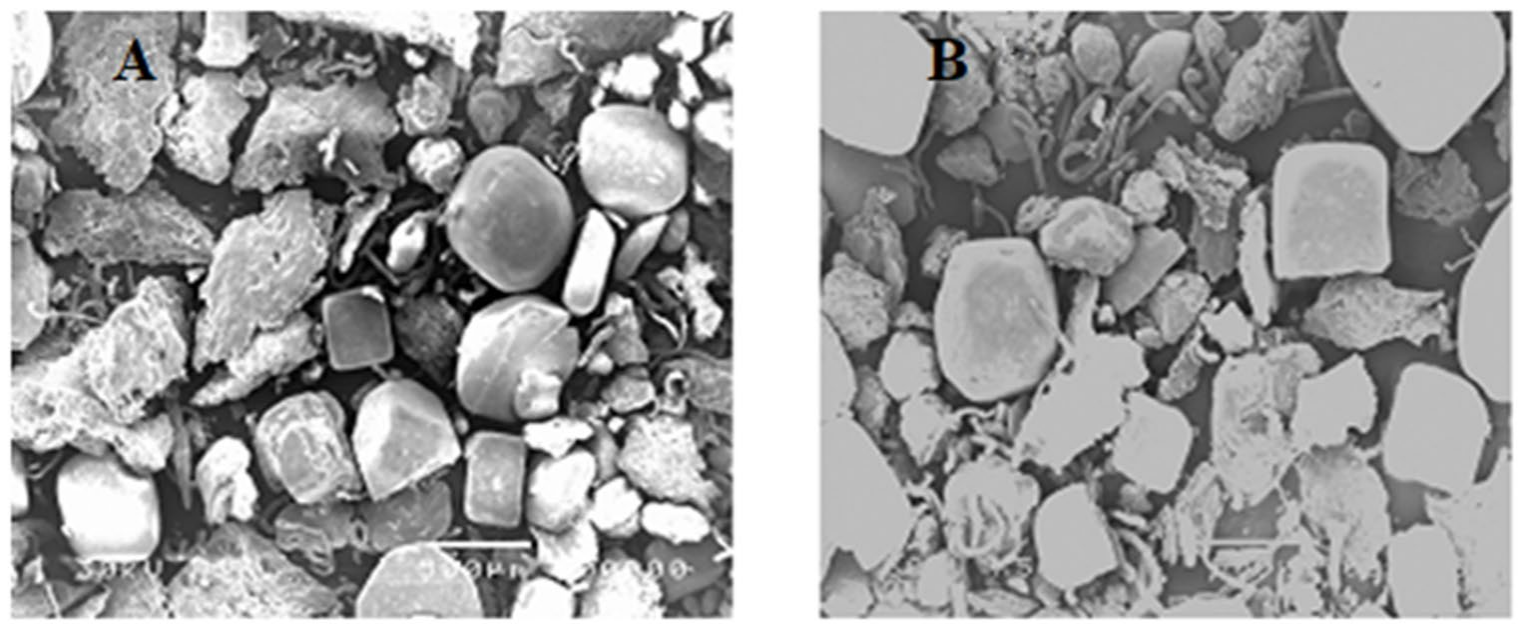
Scanning electron Microscopy (SEM) of un-irradiated (**A**) and irradiated (100 KGy) sodium alginate (**B**)

**Fig. 4:**
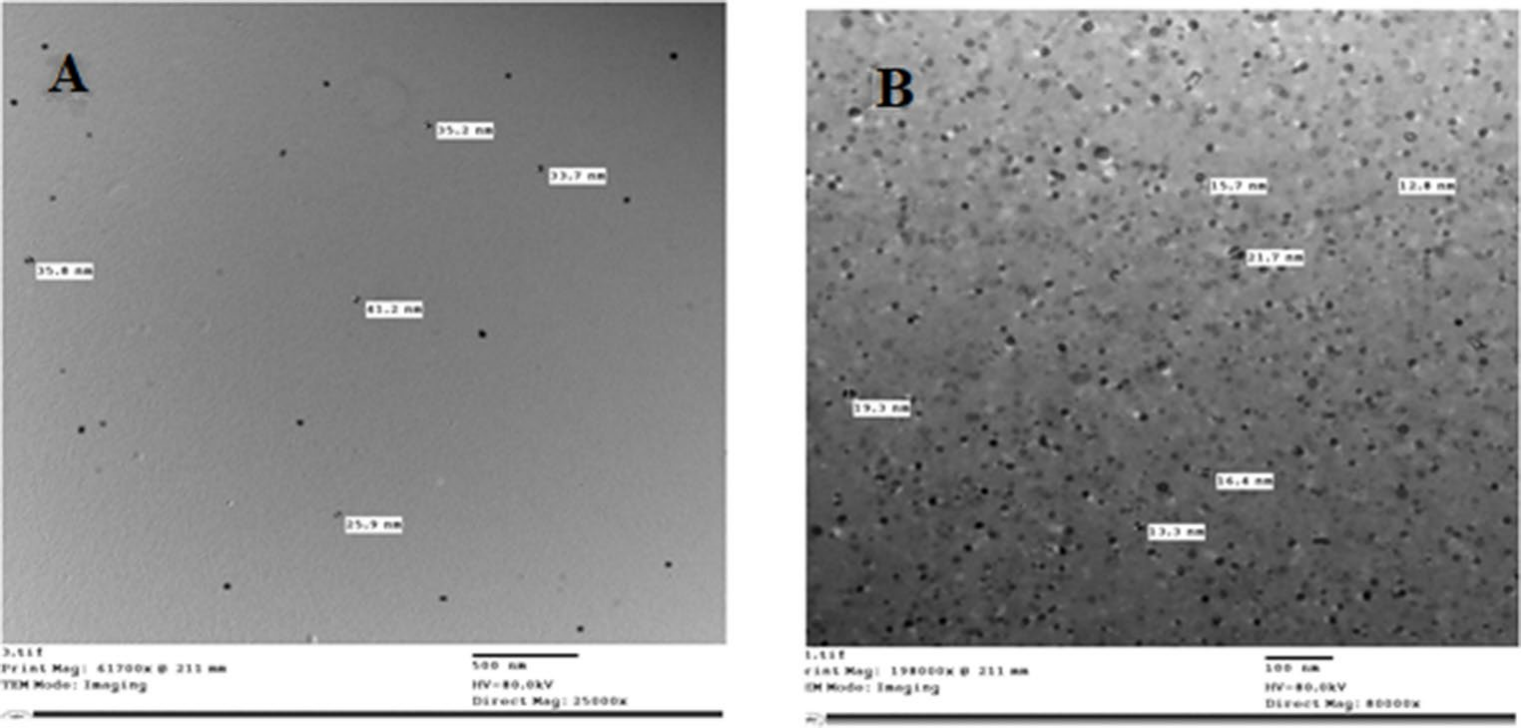
Transmission electron microscopy (TEM) of un-irradiated (**A**) and irradiated (100 KGy) sodium alginate (**B**)

The appearance of both UISA and ISA was evaluated through the use of SEM, and the surface structure was investigated with the assistance of high-resolution scanning electron microscopy. Images obtained from SEM distinct enlargement are displayed in Fig. 3. Scanning electron microscope examination revealed a reduction in particle size of irradiated sodium alginate. The results of the SEM investigation indicated that, un-irradiated sodium alginate particle size range was much bigger than irradiated sodium alginate by dose level 100 kGy. In connection to TEM (Fig. 4) UISA and ISA (100 kGy) appeared a range of the particle size between 25.9 and 41.2 nm for UISA and an average diameter of 33.55 nm, while it was12.8 to 21.7 nm for ISA and an average diameter of 17.25 nm. Current findings are in harmony by Shabbir et al. [32], who demonstrated that the pore size of sodium alginate reduced by increasing the irradiation dose up to 100 kGy, the macro-molecular structure disappeared, and the surface became smoother. Furthermore, the result was verified by SEM and XRD analysis report indicated a reduction in particle size and radiation-mediated degradation of polymers into oligomers through the breakdown of intermolecular hydrogen bonds [28]. The outcomes of the current study are in accordance with Bambal et al. [33]. It was observed that with increasing the gamma irradiation dose level the surface of the alginate particles size is gradually reduced [34].

### Quality changes in different samples

#### Weight loss

**Fig. 5:**
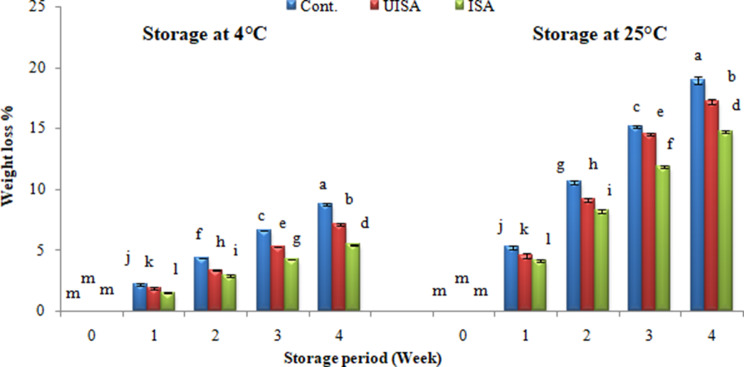
Weight loss percentage for control, un-irradiated (UISA) and irradiated (100 KGy) sodium alginate (ISA) coated treatments stored at 4, and 25 °C. Bars ± SD (*n* = 3). Different letters indicate statistically significant differences at *P* ≤ 0.05

Weight loss is due to loss of water, and this is a crucial aspect to evaluate the quality of the fruits. Furthermore, water loss has a significant impact on the appearance of the fruits [35]. The weight losses in the different samples during storage are shown in Fig. 5. The control groups experienced a more noticeable weight loss at 4 and 25 °C when compared to the tomatoes coated with sodium alginate. The weight loss of the samples after one week at 4 °C for the control group was 2.23%, while the weight loss for the un-irradiated and irradiated sodium alginate (100 kGy) treatments was 1.88 and 1.52%, respectively. At the end of the storage period the weight loss were gradually increased for the control, un-irradiated, and irradiated sodium alginate to 8.77, 7.10, and 5.46%, respectively. After one week at 25 °C weight loss of the untreated sample was 5.23% while for the un-irradiated and irradiated sodium alginate treatments was 4.55 and 4.10%, respectively. After that, the weight loss increased to 18.93, 17.20, and 14.72% for the control, un-irradiated, and irradiated sodium alginate samples, respectively, at the end of the storage period. The current findings demonstrated that the weight loss of the irradiated alginate-covered samples was less in comparison to the un-irradiated sodium alginate treatments and controls. Alginate as an edible coating can delay the fruit’s deterioration because the coating can reduce respiration and transpiration, resulting in the lowest weight loss percentage. The recent outcomes are consistent with Razali et al. [3], who exposed cherry tomatoes to UV radiation and eatable coating (mucilage of white dragon fruits), both as a stand-alone and hurdle treatment, and found that the hurdle treatment extended shelf life for 21 days and decreased weight loss. Temperature and relative humidity of the environment are important due to the effects on vapor pressure difference between fruit and atmosphere. The decrease in moisture loss of fruits coated with alginate compared with those uncoated is obviously because of the higher water vapor barrier properties associated with the coatings. Vapor-phase diffusion driven by a gradient of water vapor pressure between the fruit and the surrounding air is the primary mechanism of moisture loss from fresh fruits and vegetables [18]. Likewise, Bal et al. [36] reported that sodium alginate reduces external change and water loss by acting as a protective fence among the fruits and their environment. In the same concern, Sucharitha et al. [37] studied the impact of chitosan coating on the physical and chemical parameters of tomatoes through cold storage and found that weight loss obtained in untreated samples was attributed to fruit shrinkage as a result of moisture loss, which was not detected in fruits covered with chitosan, as the chitosan coating prevents moisture evaporation from coated tomatoes. In fact, in the process of respiration, the commodity generates carbon dioxide, water, and heat. The resulting water usually remains within the tissue. In addition, carbon dioxide escapes and accounts for part of the weight loss of the harvested organs as a result of the mass balance between oxygen intake and carbon dioxide release [38]. Weight loss causes shrinkage of the fruit exocarp and thus decreases the nutritional value, which leads to economic loss. In addition, water loss is closely related to several metabolic activities that occur after harvest and during storage [39]. Coating is a semi-permeable barrier which that reduces the direct contact between air and the surface of fruits and vegetables. Thus, coating can reduce the water loss, color change and keep the firmness of fruits and vegetables. Also, the composite or single coating solution on the surface of fruits and vegetables can maintain the quality of fruits and vegetables by resisting microorganisms, reducing gas exchange, and affecting enzyme activity [19]. Polysaccharide-based coatings modulate the internal tissue atmosphere once they exhibit an obstacle to moisture, O_2_, CO_2_, and volatiles movement, slowing down the metabolism and delaying fruit senescence [40].

#### Decay percentage

Tomato is more prone to postharvest fungal as well as bacterial decay because of higher water activity, favorable pH, and nutrient richness that serve as growth factors for microorganisms [12].

**Fig. 6:**
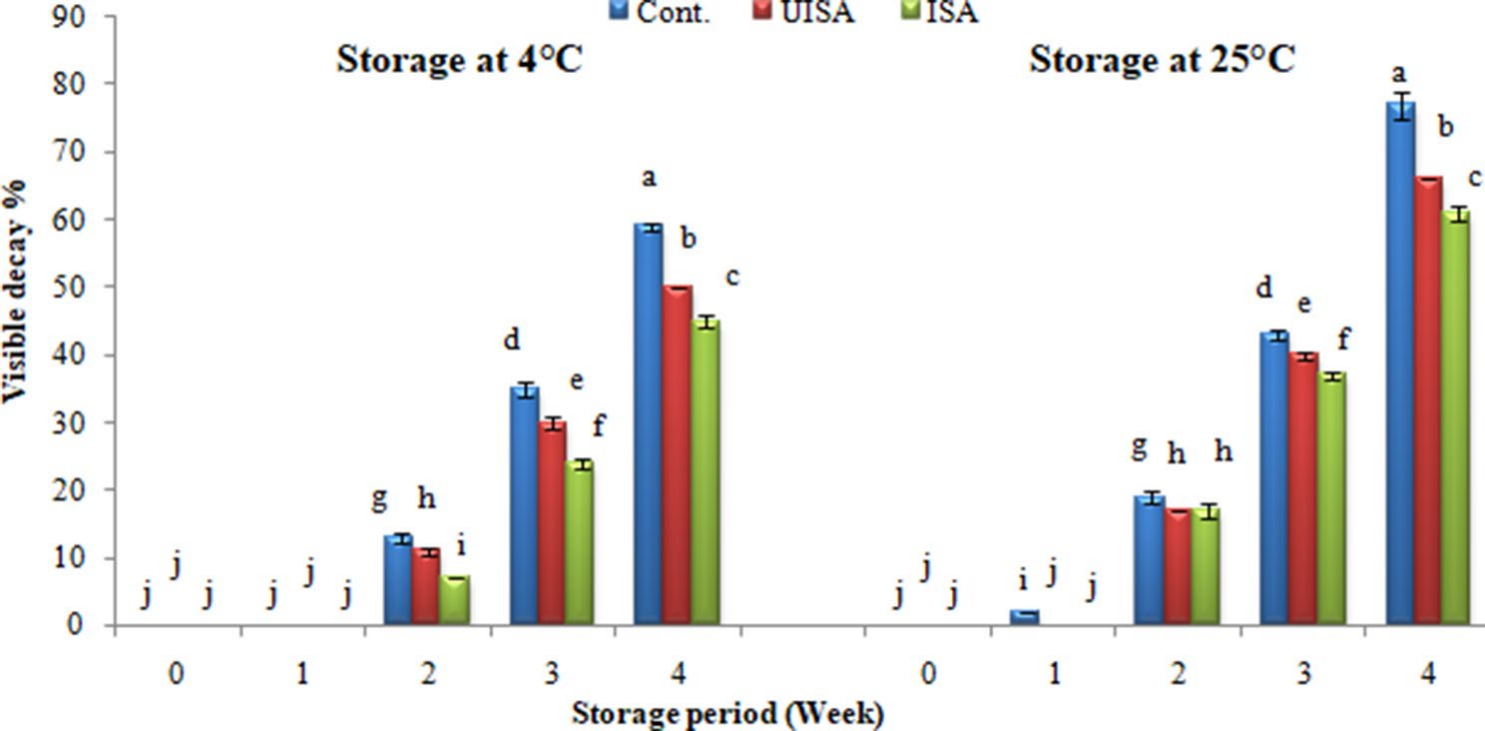
Decay percentage for control, un-irradiated and irradiated sodium alginate (100 KGy) coated treatment at stored 4, and 25 °C. Bars ± SD (*n* = 3). Different letters indicate statistically significant differences at *P* ≤ 0.05

The evolution of the incidence of decay in coated cherry tomatoes stored at 4 and 25 °C was reduced relative to untreated fruits (Fig. 6). The incidence of samples decay at 4 and 25 °C after one week was 0% for all samples except for the control sample at 25 °C, the decay was 2%. Then decay progressively increased to 13.0, 11.0, and 7.0% for the control, UISA, and ISA (100 kGy) samples, respectively, after 2 weeks of keeping time at 4 °C. Whereas, the decay at the end of storage time reached 59, 50, and 45% for the control, UISA, and ISA samples, respectively. While the decay of the treatments at 25 °C after 2 weeks was 19% for the untreated-sample, although the decay of the un-irradiated and irradiated sodium alginate samples was 17%. The decay at the end of storage time reached 77, 66, and 61% for the control, UISA, and ISA, respectively. The results indicated that decay of the UISA and ISA coated treatments was lower than the control treatments. Comparing tomato fruits coated with chitosan-*Ruta graveolens* essential oil to uncoated fruit sample, Peralta-Ruiz et al. [41] found that, the coated tomatoes significantly delayed the decay index when stored at 4 °C. It is commonly recognized that coatings based on chitosan reduce free radical presence, increase the disease resistance, and for their elicitor activity, induce the production of defense-related enzymes in fruits [42]. Eatable coating protects food products from microbial contamination, prolongs shelf life, decreases deterioration effects, and reduces lipid oxidation and moisture loss [43]. The coating reduces deterioration that may occur in the fruit due to discoloration and thus maintains the freshness of the fruit, which helps maintain the visual appearance of fruits and vegetables, which is an essential factor in consumer acceptance [44]. Storage greatly sped up ripening and senescence for tomatoes without coating. This may be due to the high rates of respiration and ethylene production turned on ripening and senescence, thus contributing to the relatively short shelf life of uncoated fruits. Coating decreased the content of oxygen and elevated carbon dioxide, which reduced respiration and delayed ethylene production; hence, ripening, senescence, and microbial development [45]. Eatable polymer coatings are considered an important technique as they have a significant function since they are regarded as an effective, secure method for conservation, leave no residue, are friendly to the environment, and can significantly avoid moisture and aroma reduction as well as inhibit the oxygen penetration to the plant tissue or microbial growth [46].

#### Fruit firmness

Firmness is a quality parameter that will affect product acceptance by consumers. Texture is a critical factor in the quality of fruits and represents metabolic changes and water content variations and is directly related to increasing the storability potential and the resistance to mechanical injury and decay [47].

**Fig. 7:**
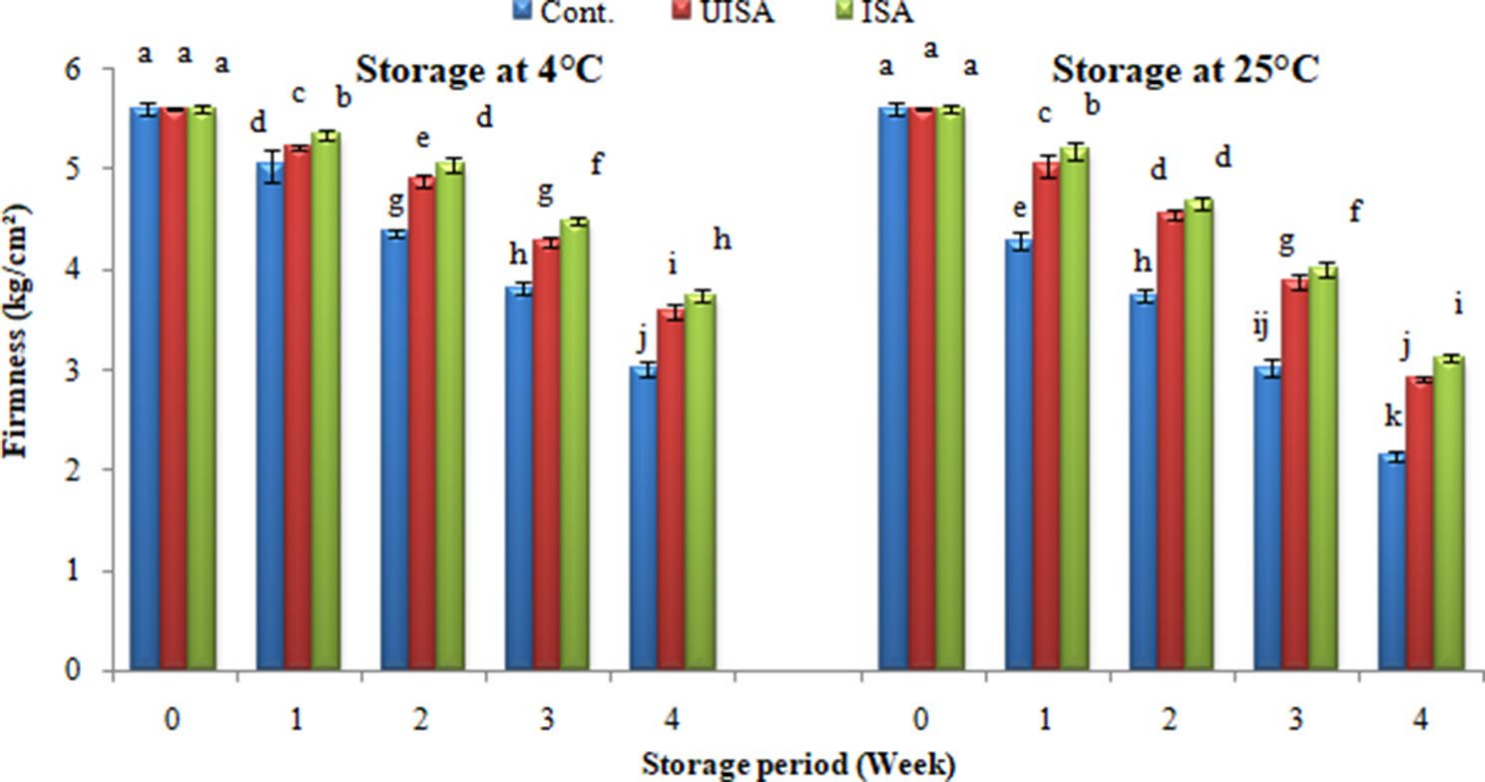
Firmness (kg/cm^2^) for control, un-irradiated and irradiated sodium alginate (100 kGy) coated samples stored at 4, and 25 °C. Bars ± SD (*n* = 3). Different letters indicate statistically significant differences at *P* ≤ 0.05

As provided by Fig. 7, the changes in cherry tomato fruit hardness values at various storage temperatures. Firmness values of the cherry tomatoes declined, demonstrating texture softening as the length of storage progressed for both the coated and uncoated fruits stored at different temperatures (4 and 25 °C). Fruits coating improved firmness maintenance significantly, with irradiated sodium alginate (100 kGy) surpassed by un-irradiated sodium alginate at both storage temperatures. The firmness of the un-irradiated sodium alginate coated fruits decreased from 5.61 to 5.23 kg/cm^2^ after one week and finally reduced to 3.59 kg/cm^2^ at the end of storage time, viewing no indication of spoilage; likewise, the firmness of the irradiated sodium alginate coated samples declined from 5.61 to 5.35 kg/cm^2^ after one week and finally reduced to 3.74 kg/cm^2^ at the end of storage period, showing no indication of spoilage when compared with untreated sample (control) wherever the firmness declined from 5.61 to 5.04 kg/cm^2^ after one week and finally to 3.01 kg/cm^2^ at the end of the storage period at 4 °C.

Likewise, at 25 °C, the firmness of the UISA-coated fruits decreased from 5.61 to 2.91 kg/cm^2^ and decreased to 3.12 kg/ cm^2^ for ISA-coated fruits at the end of storage time compared with untreated samples, wherever the texture decreased from 5.61 to 2.15 kg/cm^2^ at the same time. Higher retention of firmness applied with SA coating may be due to lower enzyme activity in the cell wall, which results in a slower rate of degradation [36]. The current findings are consistent with Annisa et al. [48], who found that the strawberries color, hardness, total soluble solid, and weight loss through refrigeration temperature storage were significantly affected by the edible coatings based on sodium alginate and various concentrations of essential oil from the siam pontianak tangerine peel. Likewise, Attar et al. [38] reported that chitosan-coated pistachio fruits had a harder kernel than uncoated fruits at the end of the storage period. Fruits will get softer as their shelf life increases because of respiration and transpiration induced wilting [49]. The existence of the processes of respiration and transpiration causes the fruit to lose water due to reduced carbon; if the water in the cell is reduced, it results in a decrease in pressure, resulting in a decrease in the value of fruit hardness, namely the fruit becomes soft and limp. The application of edible coating can maintain the hardness of fruits because the edible coating is able to withstand the migration of water from the fruit to the environment [48].

Furthermore, it is anticipated that fruit coatings will alter the interior gas composition of the fruits, particularly by raising the concentration of carbon dioxide and decreasing the concentration of oxygen, which may explain the slower textural changes in the coated fruits [50].

Therefore, edible coatings can extend the shelf life of food to a certain extent. Because the coatings can form a semi-permeable barrier on the surface of fruits and vegetables, regulate transpiration and respiration, as well as have a certain inhibitory effect on microorganisms [51].

#### Total soluble solids (°Brix)

**Fig. 8:**
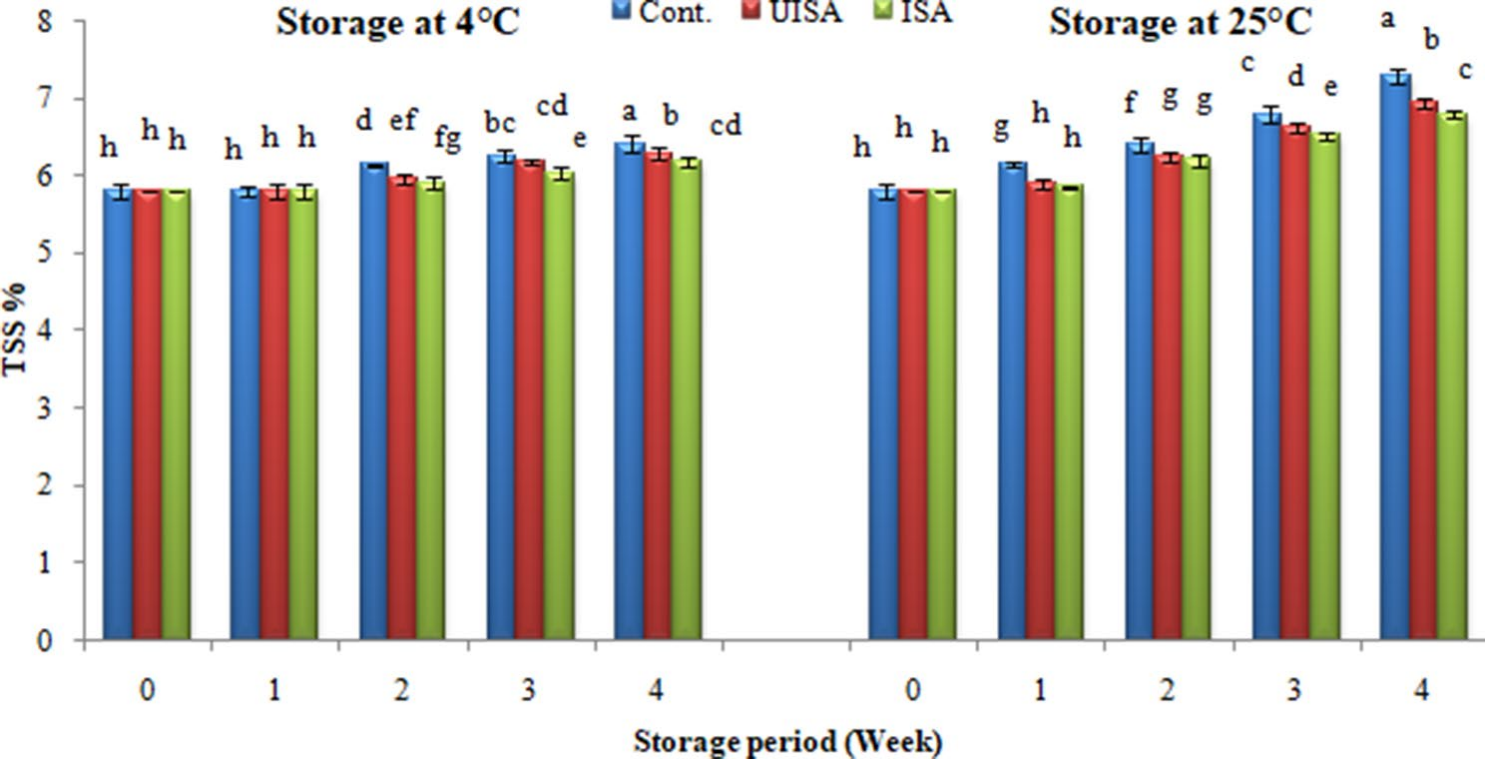
Total soluble solids percentage (°Brix) for control, un-irradiated and irradiated sodium alginate (100 KGy) coated treatments stored at 4 and 25 °C. Bars ± SD (*n* = 3). Different letters indicate statistically significant differences at *P* ≤ 0.05

Total dissolved solid percentage, or °Brix, is a crucial fruit ripening indicator, and eatable coatings are effective in lowering TSS, or, in other words, lowering ripening rates. There was a slow increase in TSS during the storage period in all treatments, but this increase in TSS for the control was higher than that of the coated samples. The total soluble solids of the untreated sample increased from 5.6 to 5.8 °Bx after 2 weeks, and maintained 6.2 °Bx till the end of the storage period at 4 °C, whereas the total soluble solids for the un-irradiated sodium alginate treatments increased from 5.6 to 5.7 °Bx and maintained 5.9 °Bx until the end of storage time. Also, the total soluble solids of the irradiated sodium alginate (100 kGy) treatment increased from 5.6 to 5.7 °Bx and maintained 5.8 °Bx until the end of storage time (Fig. 8).

At 25 °C, the total soluble solids of the untreated group increased from 5.6 to 7.0 °Bx throughout a 4-week period, while the total soluble solids improved from 5.6 to 6.6 °Bx for un-irradiated sodium alginate-coated tomatoes and 6.4 °Bx for irradiated sodium alginate coated tomatoes. The recent data are consistent with Razali et al. [3], who subjected cherry tomatoes to UV-C radiation and edible coating (mucilage of white dragon fruits) and found that the total soluble solids increased from day 0 to day 7 and decreased after that. On the other hand, Mannozzi et al. [52] reported that the soluble solid content of blueberries coated with alginate did not exhibit any significant variation. Also, Maurizzi et al. [53] found that the soluble solid concentration of the treatments rises with time, and this impact is related to the fruits ripening. During this process, resulted enzymes are responsible for the conversion of complex polysaccharides into simple sugars, which are sources of the respiration system that ultimately leads to fruit senescence. In tomatoes, phosphorolytic and hydrolytic enzymes contribute to raising the total soluble solids levels by shifting the balance from starch synthesis to degradation [54]. Previous findings showed that the increase in the ripening index was limited by the coated fruits through storage. The coated fruits had less accumulated TSS because of the coating reduced the respiration rate and metabolic activity by blocking the gaseous exchange, delaying the ripening process, and limited moisture loss [35, 55]. The increased amount of soluble solids in the untreated fruits (control) is attributed partly to water loss and drying of cherry tomato fruit; however, the breakdown of the complex carbohydrate to soluble sugars through normal maturity is directly responsible for the increase in total soluble solids, which is also observed as pulp softening. Also, the maturity of cherry tomatoes is caused by the degradation of organic acid and the accumulation of sugars during storage [56].

#### Titratable acidity (as citric acid percentage) and pH value

The titratable acidity is a measurement of fruit’s organic acid content (as citric acid %), which decreases during postharvest storage as a result of using the organic acids as substrates for respiratory metabolism [35]. Titratable acidity decreased in all samples over time during storage at both temperatures, and the decreasing rate was fast at 25 °C and slow at 4 °C. However, the titratable acidity of coated samples was higher compared to the control treatment.

**Fig. 9:**
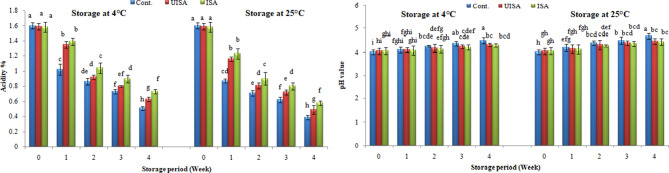
Titratable acidity (as citric acid percentage) and pH value for control, un-irradiated and irradiated (100 KGy) sodium alginate coated samples stored at 4, and 25 °C. Bars ± SD (*n* = 3). Different letters indicate statistically significant differences at *P* ≤ 0.05

At 4 °C, the titratable acidity of the coated cherry tomatoes with the un-irradiated alginate decreased from 1.59 to 0.63% at the end of storage time, as well, the acidity of the irradiated sodium alginate treatments (100 kGy) decreased from 1.58 to 0.73% at the end of the storage period, whereas the acidity of the untreated treatment (control) reduced from 1.60 − 0.51% (Fig. 9).

At 25 °C, similar trends were observed, and it could be seen that the decreasing rate of acidity was faster at 25 °C than at 4 °C, even though the acidity of the coated samples was higher than the control. The titratable acidity of the coated cherry tomatoes with the un-irradiated alginate decreased from 1.59 to 0.49% at the end of storage time, and the acidity of the irradiated sodium alginate treatments (100 kGy) decreased from 1.58 to 0.58% at the end of storage period, while the acidity of the untreated sample (control) reduced from 1.60 to 0.39%. The coatings were able to minimize the ethylene production in coated cherry tomatoes, thereby maintaining acidity compared to the untreated treatment. These results are in the same pattern with Wu et al. [57], who investigated the effects of sodium alginate-based edible coatings with `Baozhu` pear chitinase on the quality of cherry tomatoes through cold storage and found that these coatings might inhibit weight loss, maintain hardness, and slow down titratable acidity changes and vitamin C. Also, Bal [58] indicated that titratable acidity of tomatoes decreased overtime compared to the initial value (0.52%) varying between 0.40% (UV-C) and 0.44% (alginate) at the end of the storage period. According to Xing et al. [59] and Yang et al. [60], the reason for the decrease in total acidity content was due to the acids, which are the major substances of respiratory metabolism. Alginate coating could delay the changes in acidity by reducing the respiration rate [61]. Organic acids are substrates for several enzyme catalyzed reactions during aerobic respiration in plant cells, and the acidity reduction may be expected as a result of such activity during the ripening process, thus making the fruits taste relatively sweeter. The reduction in the acidity reveals advancement of maturity of fruits/ripening; hence coated fruits help to postpone ripening. So, the decrease in acidity reflected the development of maturity of the fruit during storage in the refrigerator. On the other hand, high titratable acidity may also be the reason for the creation of carboxylic acids by dark fixation of carbon dioxide [62].

Coated fruits had comparatively less change in their pH value because they displayed fewer variances in the titratable acidity. The pH value of the coated fruit was always lower than that of the untreated cherry tomatoes (Fig. 9). The increasing level of pH value in untreated treatment (control) was slightly more than in coated fruits. The irradiated alginate coating treatments retained pH value better than the un-irradiated sodium alginate coating samples.

The pH of the un-irradiated and irradiated alginate-coated samples was lower than the control cherry tomatoes at both storage temperatures (4 and 25 °C). The edible coatings had a protective effect of reducing the ripening rate of the fruits by maintaining the acidity of the fruits compared to the control. The pH values of all the samples irrespective of the control eventually increased with time at both temperatures, and the increasing rate was fast at 25 °C, whereas the increasing rate was slow at 4 °C.

At 4 °C, the pH value of the un-irradiated alginate samples increased slightly from 4.05 to 4.18 and from 4.06 to 4.14 for irradiated samples after 2 weeks, whereas the pH value of the untreated treatment (control) increased gradually from 4.01 to 4.26 (Fig. 9). Similar trends were noticed at 25 °C, the pH value slightly increased from 4.04 to 4.31 for un-irradiated sodium alginate treatments and from 4.07 to 4.27 for irradiated sodium alginate treatments after 2 weeks, whereas the pH value of the untreated sample increased gradually from 4.02 to 4.39 at the same time. The pH value increasing rate at 25 °C was higher than 4 °C. The pH values of all the alginate-coated treatments were low, indicating that sodium-alginate coatings postponed biochemical reactions due to ripening [63]. Moreover, Maurizzi et al. [53] applied hydroxypropyl methylcellulose (H)-guar gum and potassium sorbate as coatings on tomatoes and oranges, as well as observed that there was an increase in the acidity of all treatments during the storage period depending on the type of coating. The pH parameter is indirectly correlated to the titratable acidity values of the fruit juice during post-harvest storage [64]. As a result of the fruits and vegetables metabolism, the contents of polysaccharide change during maturation owing to acid hydrolysis, as well as these alterations might be reflected physiological and biochemical changes addition to post-harvest decompose   [65].

### Content of lycopene in cherry tomatoes

**Fig. 10:**
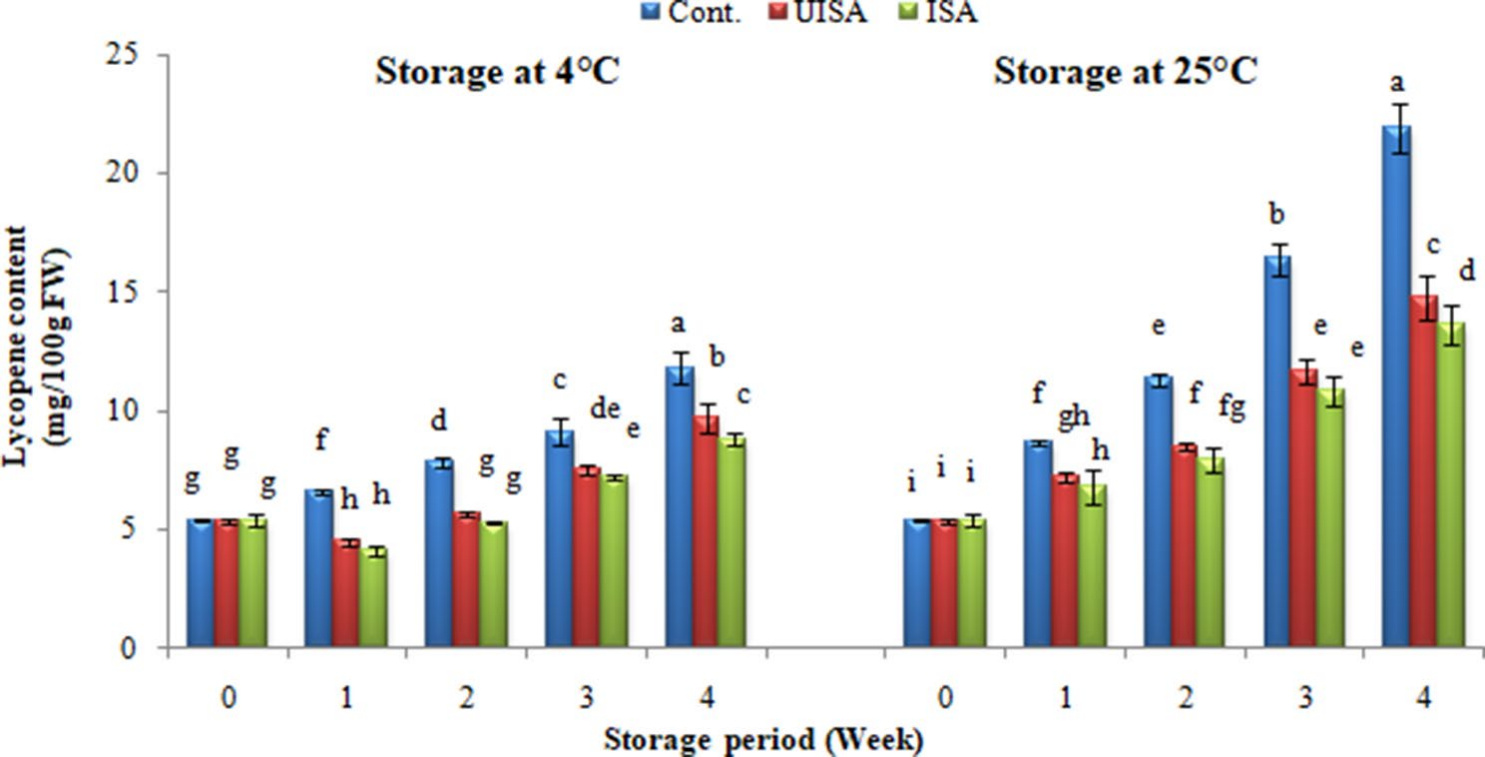
Content of lycopene for control, un-irradiated and irradiated sodium alginate (100 kGy) coated samples stored at 4, and 25 °C. Bars ± SD (*n* = 3). Different letters indicate statistically significant differences at *P* ≤ 0.05

The primary pigment that gives tomatoes their characteristic red color is called lycopene, and it is one of the carotenoids that are most commonly found in tomatoes. The content of lycopene varies widely among tomato varieties and increases dramatically during ripening [66]. The data in Fig. 10 revealed that lycopene contents were steadily raised as the storage time increased, where the smallest values occurred at the beginning of storage (5.4 mg/100 g FW) and after that increased significantly with the prolongation of the storage time in both temperatures. At the end of the storage period, the control treatment exhibited significantly higher content of lycopene (11.80 and 21.90 mg/100 g at 4 and 25 °C, respectively) in comparison with sodium alginate (9.70 and 14.80 mg/100 g, respectively) and irradiated sodium alginate (8.80 and 13.60 mg/100 g, respectively) treatments at the same temperatures. According to Mohammed et al. [67], the increasing of lycopene content with the elapse of the storage period may be due to the production of lycopene content, which is directly correlated with ripening, and the formation of lycopene depends on the temperature range and rate of respiration during storage. The findings presented here are in concordance with the findings of Abdullah and Ibrahim [68] on cherry tomatoes. Also, Bal [58] indicated that UV-C and edible coating treatments lead to less lycopene content of cherry tomatoes compared to the non-treated. Treating cherry tomato fruits with sodium alginate (UISA and ISA) significantly reduced the lycopene value of the tomato fruit compared with an untreated sample, which resulted in a significant rise in the lycopene content. Treatment with irradiated alginate was the most effective in reducing lycopene content, which led to a decrease in lycopene content compared to control and un-irradiated alginate. The early increase in lycopene concentration in control fruit may be due to the faster ripening of fruits than in the fruits treated with SA, whilst SA treatment has beneficial effect on fruit physiology such as delaying ripening of fruits and at the same time, slowed down the respiration rate and ethylene production in cherry tomatoes.

### Total phenolic compounds & flavonoids content and antioxidant activity

**Fig. 11:**
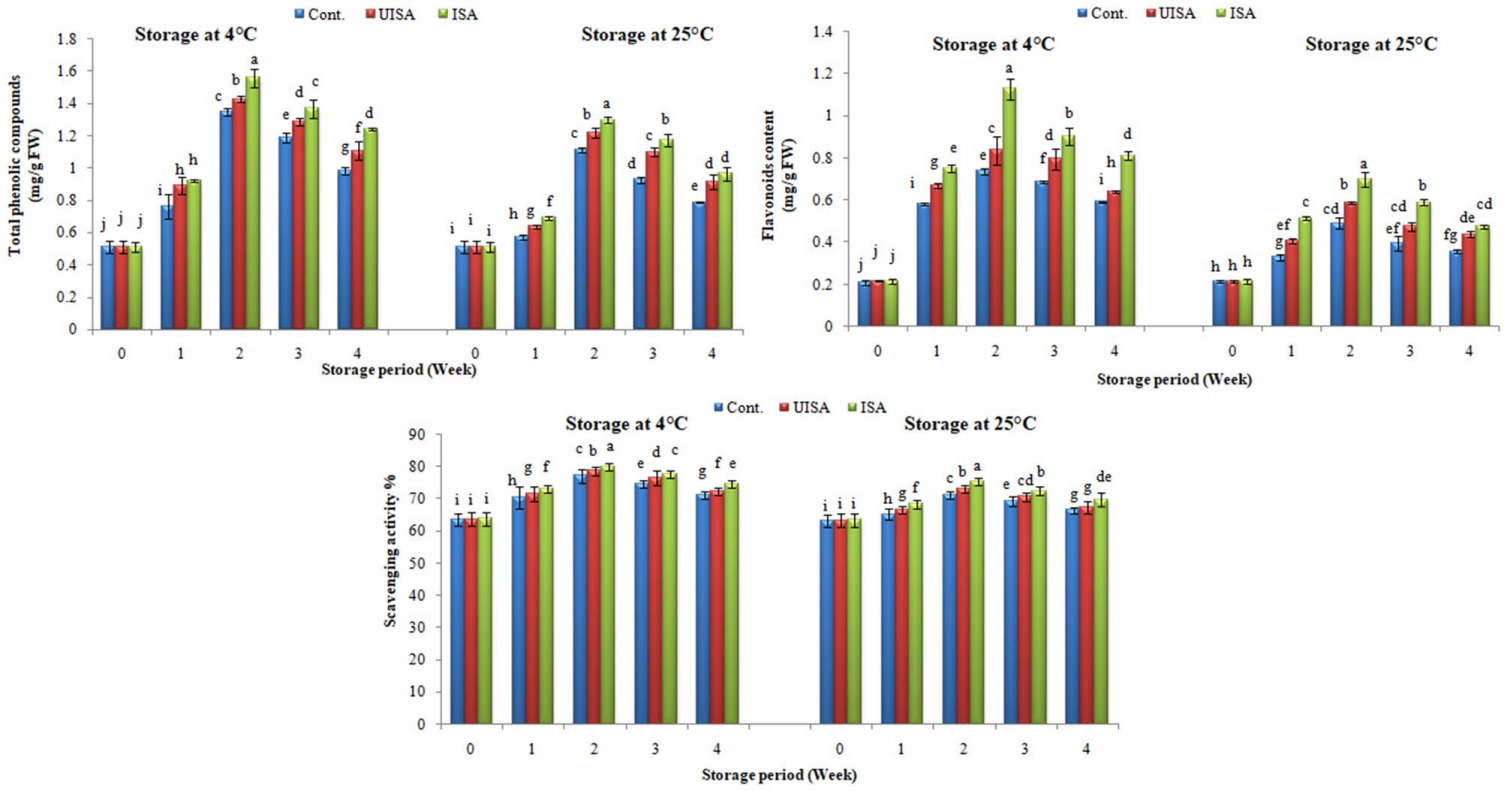
Total phenolic, flavonoids compounds (mg/g fresh weight) and scavenging activity percentage for control, un-irradiated and irradiated (100 KGy) sodium alginate coated samples stored at 4 and 25 °C. Bars ± SD (*n* = 3). Different letters indicate statistically significant differences at *P* ≤ 0.05

The total phenolic compounds expressed as mg of gallic acid per gram of fresh sample showed an increasing trend to a peak value and then reduced in all treatments at all the storage temperatures (Fig. 11). The total phenolic compounds of un-irradiated alginate samples at 4 °C had increased from 0.513 to 1.43 mg/g FW, a peak value after 2 weeks, while it was reduced to 1.11 mg/g FW at the end of the storage time. Whereas the phenolic content of untreated samples (control) is increased from 0.512 to 1.35 mg/g FW after 2 weeks and finally decreased to 0.986 mg/g FW at the end of the storage period (Fig. 11).

Similarly, the total phenolic compounds of irradiated alginate coated cherry tomatoes have been raised from 0.514 to 1.56 mg/g FW after 2-weeks, and it is reduced to 1.24 mg/g FW at the end of storage time.

The same trends in results were observed at 25 °C for the coated and control samples; the total phenolic content of un-irradiated sodium alginate increased from 0.511 to 1.22 mg/g FW after 2 weeks, while the total phenolic content reduced to 0.916 mg/g FW at the end of storage time, whereas the phenolic content of untreated samples (control) increased from 0.512 to 1.12 mg/g FW after 2 weeks and finally decreased to 0.789 mg/g FW at the end of the storage period.

Also, the total phenolic compounds of irradiated sodium alginate-coated cherry tomatoes increased from 0.513 to 1.30 mg/g FW after 2 weeks, and it was reduced to 0.967 mg/g FW at the end of the storage period.

The flavonoids content of un-irradiated alginate samples at 4 °C is raised to its maximum value after 2 weeks from 0.215 to 0.837 mg/g FW, while it is decreased to 0.640 mg/g FW at the end of the storage period (Fig. 11). Whereas, the flavonoids content of the untreated sample is enhanced from 0.209 to 0.738 mg/g FW after 2 weeks and finally decreases to 0.593 mg/g FW at the end of the storage time. Correspondingly, the flavonoids content of cherry tomatoes treated with irradiated alginate has improved from 0.213 to the greatest content of 1.13 mg/g FW after 2 weeks, while it is decreased to 0.813 mg/g FW at the end of the storage period. Same patterns are noted at 25 °C for treated and untreated tomatoes. The phenolic and flavonoids content of the irradiated sodium alginate samples was higher than that of the un-irradiated sodium alginate and control samples during storage.

These findings are in agreement with Razali et al. [3], who established that cherry tomatoes treated with an eatable coating (mucilage of white dragon fruits) combined with UV radiation contained high polyphenols and flavonoids. Also, they pointed out that edible coating acted as a potential abiotic stress on the fruit, which led to the formation of secondary metabolites such as phenolic and flavonoid compounds. Also, Khedr and Khedr [69] demonstrated that mango treated with progesterone (PROG) could effectively delay the decline of phenolic content during storage, and the total phenolic content of mango treated with PROG was higher than that of the control sample. According to Dávila-Aviña et al. [70], the formation of phenolic components during storage is enhanced when the oxygen and carbon dioxide contents are low and high, respectively. As total phenolic content was shown to be lower in untreated fruit, which is attributed to a negative correlation with weight loss and decay percentage. The use of alginate has been shown to enhance the phenolic contents of fruits and thus increase their quality [36]. The recent results are in accordance with Martinez-Espla et al. [71], who indicated that SA application enhanced total phenolic content (TPC) accumulation during the storage due to activation of phenylalanine ammonia layase (PAL), a key enzyme of the phenylpropanoid pathway. But the decrease of phenolic compounds during storage may be due to the breakdown of cell structure as a result of aging during ripening, and the activity of polyphenol oxidase and the synthesis of phenolic compounds are generally affected by various biotic and abiotic stresses, including chilling [69].

Regarding the antioxidant activity, Fig. 11 shows the DPPH radical scavenging activity in tested samples during storage. The antioxidant activity of all tested samples increased and reached a maximum value after 2 weeks and then decreased at the end of the storage time for both temperatures (4 and 25 °C). The antioxidant activities of the irradiated sodium alginate treatments were greater than un-irradiated sodium alginate and untreated treatments (control) for both 4 and 25 °C. The application of edible coatings to fresh fruits has been associated with an accumulation of phenolic compounds, resulting in an increase in the fruit’s antioxidant capacity [72].

The use of 1-(3-phenyl-propyl) cyclopropene or melatonin as anti-ethylene agents was found to effectively maintain the postharvest quality parameters, in terms of bioactive compounds, energy metabolism, and antioxidant activity, of mango fruits stored at 5 ± 1 °C for 28 days [39].

Owing to the impact of phenolic compounds on the flavor, color, aroma, and taste of fruits, these compounds play a vital role in the fruits goodness. As a result, the treated fruits which contain high phenols would have greater quality than the untreated fruits. Sodium alginate coatings of fruits conserved these components throughout the storage time via delaying the ripening process [73].

The obtained findings were in agreement with an investigation by Rastegar et al. [74], who established that decreasing the phenol level of mango fruits was inhibited with alginate-based edible coating application. Further study has pointed out that the coating fruits like strawberries with sodium alginate resulted in a drop in total phenol loss [75]. Concerning the role of the antioxidants in human health, the antioxidant content of the products is one of the important factors in the quality of fruits. Generally, increasing reactive oxygen species (ROS) production during fruit storage leads to oxidative stress, resulting in the degradation and decay of products [74].

Plants have changed both enzymatic and non-enzymatic antioxidant systems in order to regulate ROS formation through physiological processes. The decrease in the total antioxidants during product storage may be due to oxidation of phenolic compounds and ascorbic acid [76]. Based on the findings of González-Aguilar et al. [72], it is believed that edible coatings have the ability to affect the internal atmosphere, resulting in a delay in the fruit’s metabolism. It also helps restrict the changes in phenols and flavonoids concentrations, which in turn leads to the accumulation of bioactive compounds, resulting in increasing the antioxidant activities. Same trends have been obtained by Takma and Korel [77], who observed that alginate coatings supplemented with vanillin were able to maintain the primary antioxidant activities of grapes stored for 35 days at 4 ± 2 °C.

### Water-soluble vitamins content in cherry tomatoes

The result shown in Table 2 reveals that the content of water-soluble vitamins throughout the storage time at 4 and 25 °C tends to decrease. Vitamins loss in cherry tomatoes has been affected by a number of variables, counting oxygen, temperature, light, pH value, water activity, and enzymatic modification. It is known that water-soluble vitamins are more susceptible to leach loss, while vitamin C is also more susceptible to chemical oxidation. The degree of vitamin destruction depends on the type of vitamin, the length of processing, and the storage period [78]. Over time, the majority of vitamins found in cherry tomatoes, especially vitamin C, have decreased (Table 2). The primary concentration of vitamin C in cherry tomatoes was 138.62 mg/kg FW and decreased at the end of storage time to 129.77 and 133.13 mg/kg FW for both un-irradiated and irradiated sodium alginate, respectively, at 4 °C, while its concentration was 73.65 and 83.30 mg/kg FW, respectively, at 25 °C, compared to the control (121.68 and 67.03 mg/kg FW), respectively. Among water-soluble vitamins in cherry tomato samples was riboflavin (vitamin B2), which had a content of 0.927 mg/kg FW at the beginning of storage and then decreased significantly at the end of the storage period to reach 0.764 and 0.842 mg/kg FW at 4 °C for both UISA and ISA treatments, respectively, while its content at 25 °C reached 0.351 and 0.368 for both UISA and ISA treatments, respectively. Similarly, niacin (vitamin B3) declined gradually with storage time to 5.90 and 5.96 mg/kg FW for both un-irradiated and irradiated sodium alginate treatments, respectively, at 4 °C, while its content at 25 °C was 5.19 and 5.61 mg/kg FW for both UISA and ISA treatments, respectively, compared to its content at the beginning of storage (7.08 mg/kg FW).

The content of pyridoxine (vitamin B6) and folate (vitamin B9) was also affected by storage, especially at 25 °C (Table 2). Their content at the beginning of storage was 3.85 and 0.526 mg/kg FW, respectively, then reached to 3.59 and 0.465 mg/kg FW, respectively, for the UISA treatment and 3.75 and 0.471 mg/kg FW for the ISA treatments at 4 °C, and the same trend occurred at 25 °C. These results are consistent with Nguyet et al. [78], who found that almost water-soluble vitamins (B1, B5, B6, B9, and C) in *Sauropus androgynous* leaves tend to lose around 50% of their weight after 8 days of storage. Likewise, Yao et al. [15] indicated that freshly cut lettuce samples treated with carbon dots/chitosan reduced oxygen exposure, which prevented the oxidation process of ascorbic acid, which helped maintain the ascorbic acid content in cut lettuce.

From this, it is clear that tomatoes treated with sodium alginate showed the least reduction in the content of water-soluble vitamins, especially irradiated sodium alginate treatment compared to the control. Vitamin C content in tomato fruits depends on the genotype, climatic condition, fruits growth, ripening, aging, and preservation period, and its content in tomato fruits increases to a maximum and then begins to decrease with ripening [58].

Decreasing in vitamin C throughout storage time might be attributed to the action of ascorbic acid oxidase, which converted ascorbic acid to dehydroascorbic acid [79]. The use of edible coating causes low oxygen permeability followed by decreased enzyme activity, resulting in a reduction of ascorbic acid oxidation [35]. The outcomes of the recent investigation were in line with those obtained by Bal [58], who reported a significant retention of vitamin C in coated tomato fruits.

**Table 2:** Water-soluble vitamins (mg/kg FW) for cherry tomatoes at the beginning and after four-weeks of storage time

Vitamins	Zero time	After four weeks					
		Storage at 4 °C			Storage at 25 °C		
		Cont.	UISA	ISA	Cont.	UISA	ISA
Vit. C (ascorbic acid)	138.62 ± 2.9^a^	121.68 ± 4.1^c^	129.77 ± 4^b^	133.13 ± 2.6^b^	67.03 ± 4.3^f^	73.65 ± 3.9^e^	83.30 ± 3.4^d^
Vit. B2 (riboflavin)	0.927 ± 0.03^a^	0.656 ± 0.05^d^	0.764 ± 0.07^c^	0.842 ± 0.10^b^	0.315 ± 0.03^e^	0.351 ± 0.03^e^	0.368 ± 0.01^e^
Vit. B3 (niacin)	7.08 ± 0.13^a^	5.37 ± 0.09^d^	5.90 ± 0.08^b^	5.96 ± 0.09^b^	3.73 ± 0.07^f^	5.19 ± 0.04^e^	5.61 ± 0.03^c^
Vit. B6 (pyridoxine)	3.85 ± 0.15^a^	2.98 ± 0.12^c^	3.59 ± 0.13^b^	3.75 ± 0.22^ab^	2.15 ± 0.09^d^	3.06 ± 0.09^c^	3.17 ± 0.06^c^
Vit. B9 (folate)	0.526 ± 0.007^a^	0.424 ± 0.009^b^	0.465 ± 0.006^b^	0.471 ± 0.004^b^	0.236 ± 0.004^d^	0.292 ± 0.006^c^	0.310 ± 0.005^c^

Results are given as averages ± SD (*n* = 3). Different letters in the same row indicate statistically significant differences at *P* ≤ 0.05

### Correlation heatmap between physio-chemical quality of cherry tomatoes

**Fig. 12:**
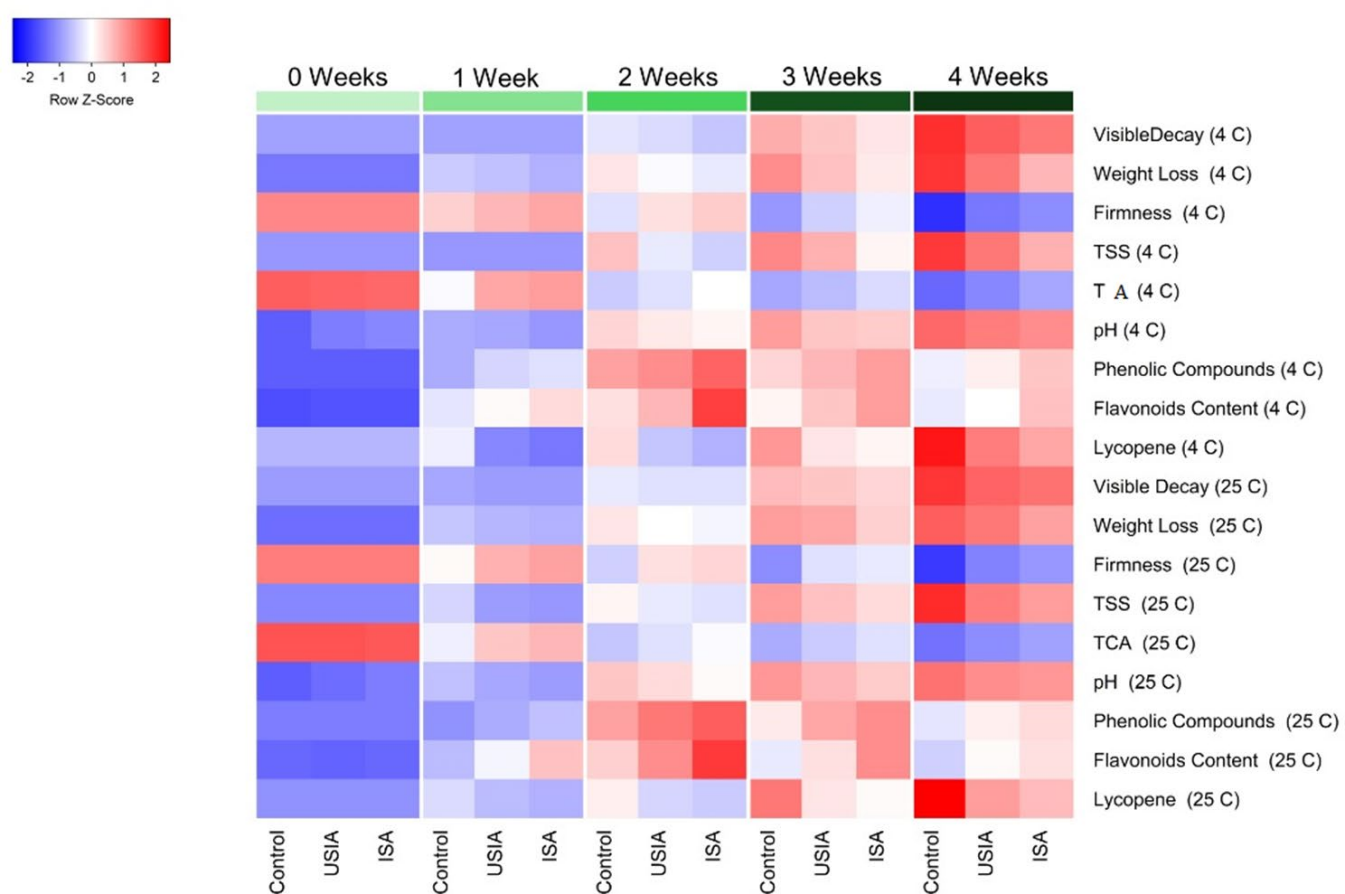
Correlation heatmap between physio-chemical quality of cherry tomatoes fruit for control, un-irradiated and irradiated (100 kGy) sodium alginate coated samples stored at 4 and 25 °C. Red color indicates a positive relationship, while blue color indicates a negative relationship

The alterations occurring in the physical and chemical characteristics of cherry tomatoes treated with SA and ISA during storage time are shown in Fig. 12. It is generally observed that there was a gradual decrease in the hardness of the fruits with increasing storage time, and the amount of weight loss, deterioration rate, and lycopene increased with increasing storage time. While the content of phenols and flavonoids increased up to two weeks of storage, after which a gradual decrease occurred with increasing storage period. At the end of the storage period, the cherry tomato fruits coated with irradiated sodium alginate gave the highest amount of fruit firmness, phenols, and flavonoids. It also reduced the loss in fruit weight, deterioration, total soluble solids, and lycopene content more than the rest of the treatments. These results revealed that treating cherry tomatoes fruit with sodium alginate, especially irradiated ones, are effective in delaying the ripening and aging process of these fruits. In addition to that, both treatments preserved the shape and quality of the fruits.

## Conclusions

The use of sodium alginate as an edible coating on cherry tomatoes resulted in the delay of the ripening process, as it had a significant effect on physical measurements such as weight loss, deterioration, firmness, and total soluble solids during the two degrees of storage (4 and 25 °C). It was noted that the fruits coated with SA contained a loss of weight less than the un-treated fruits. Also, it is clear that tomatoes treated with alginate showed the least decrease in water soluble vitamins content, especially ISA. It could be concluded that irradiated sodium alginate coatings was effective in maintaining a higher quality of the physicochemical measurements of cherry tomatoes comparison with untreated sample throughout the storage period. Additionally, there are presently very few commercial uses for alginate coatings and the majority of investigations being done at the lab level. The industrial use to commercialize food products coated with alginate and having a longer shelf life should be the main focus of future research with industrial applications.

## Materials and methods

### Plant material

Cherry tomato fruits (*Solanum lycopersicum* L. Cv. Catanya) were purchased from El- Yakout farm, Werdan, Imbaba, Giza, Egypt. Fruits were immediately transported to the laboratory, and fruits selections were made based on the uniformity of size, color, shape, and the absence of defects, injuries, and fungal infection. Selected fruits were washed in running water and sanitized with a cooled sodium hypochlorite solution (200 ppm) for 2 min and then left to dry at room temperature (25 °C) for about 1 h [80]. The fruit samples were divided according to the storage temperature into two groups, the first group was stored at a temperature of 4 °C and the second group was stored at 25 °C. Then each group was divided into three subgroups. The first subgroup constituted; the controls which were stored without coating, the second subgroup was coated with the un-irradiated alginate, and the third subgroup was coated with the irradiated sodium alginate.

### Chemical and solvents

Sodium Alginate (Sigma-Aldrich, USA), Diphenyl-2-Picrylhydrazyl (DPPH), sodium carbonate, Folin-Ciocalteu’s phenol reagent, gallic acid, aluminium chloride hexahydrate (AlCl_3_. 6 H_2_O), and quercetin were obtained from Sigma-Aldrich (Sigma–Aldrich, Milan, Italy). Sodium nitrite, ethanol HPLC grade and sodium hydroxide were supplied by Roth Company (Overland Park, KS, United States). Glycerin and calcium chloride were obtained from (El-Gomhouria chemical company, Cairo, Egypt). The other reagents that were used in each experiment were analytical grade and obtained from (Acmatic For Chemicals & Lab. Equipment Company, Cairo, Egypt).

### Irradiation treatment

Sodium alginate was degraded by subjected to gamma irradiation ^60^Co source at different dose levels (25, 50, 75, and 100 kGy). The dose rate for all samples was 1.0 kGy/h, respectively at the irradiation time. Gamma irradiation treatments have been done at the Egyptian Atomic Energy Authority, National Center for Radiation Research and Technology, Cairo, Egypt.

### Characterization of sodium alginate (SA)

#### Fourier transform infrared (FTIR) spectroscopy

Infrared spectra for un-irradiated and gamma irradiated sodium alginate were performed by the FTIR spectrometer (Bruker, Unicom, Germany) at a resolution of 4 cm^− 1^ in the 4000 –500 cm^− 1^ wavelength range [81].

#### X-Ray diffraction (XRD)

The XRD of un-irradiated and gamma irradiated SA, were measured using XD-DI Series instrument equipped (Shimadzu 6000, Japan) with Ni-filtered and Cu-K target (λ = 1.542), 30 mA electric current, 40 kV operating voltage, and a scanning angle (2*θ*) range of 4–80° at a speed of 8°/min [82].

#### The thermogravimetric analysis (TGA)

The TGA determination of un-irradiated and gamma irradiated SA, were performed by a Shimadzu–50 apparatus (Kyoto, Japan) at a flow rate of 50 ml/min of pure nitrogen gas while heating the samples from 25 °C to 600 ºC at a rate of 10 ºC/min [82].

#### Scanning electron microscope (SEM)

Samples surface were scanned with JSM-5200 SEM, Japan at voltage acceleration of 25 kV after gold deposited in vacuum for 3 min [83].

#### Transmission electron microscopy (TEM)

The SA structures were observed using TEM (JEOL - JEM 1400CX Electron Microscope, Japan) with acceleration voltage 80 kV [84].

Based on these previous characteristics, the best irradiation dose of sodium alginate (100 kGy) was chosen to be used in cherry tomatoes coating.

### Preparation and application of the alginate coating

Un-irradiated and irradiated sodium alginate (2%) was dissolved in H_2_O at 45 °C with permanent shaken till the solution became clear. After cooling to 20 °C, glycerol at 20% (v/v) was added as plasticizer.

After dipping the cherry tomatoes in sodium alginate solution for two minutes, then they were taken to net containers to remove any remaining coatings. The coated tomatoes were placed in CaCl_2_ solution (2%) to help set the coatings for two minutes. After drying for three hours at 25 °C, and packed in punntes, each punnet (250 g) which were considered as an experimental unit representing one replicate and each treatment consisted of 12 punntes.

The following assessments were made to monitor quality changes in test samples every week until the fruits showed signs of spoilage and three replicates were randomly taken.

### The weight loss percentage

The percentage of weight loss was determined using the Eq. (1) that was outlined according to Akhtar et al. [85].

Weight loss % = [(Initial fruits weight - Final fruits weight) / Initial fruits weight] x 100 (1).

### Decay percentage

The decay percentage of all tested samples was evaluated as the total number of decayed fruit showing indication of fungal appearance compared with total number of fruit initially taken and expressed as percentage [86, 87].

### Fruit firmness

Firmness (hardness or softening) was determined using a penetrometer (NEWTRY, China) which consists of a plunger which is pressed against the flesh abed when this gives way a pressure reading is given by scale in kg/cm^2^ [88].

### Total soluble solids (TSS)

Fruits TSS were evaluated by utilizing a hand refractometer (ATC Refractometer, China) to determine the refractive index of fruit juice [89].

### Titratable acidity

Five cherry tomatoes were chosen at random from each treatment, blended in an electrical blender, then filtered to produce a clear homogenate juice. The method of calculating titratable acidity involved titrating 10 ml of tomato juice, diluted to 100 ml with deionized H_2_O against 0.1 N NaOH solution. The result was stated as a percentage of citric acid. Using the formula (2) [90], .

Citric acid % = [(vol. of NaOH (in liter) x 0.1 NaOH x 64.04 / weight of the sample)] x 100 (2).

### pH value

pH value of fruits juice was measured using a pH meter 3310 (Jenway-3310, UK).

### Lycopene content

Lycopene contents were provided by protocol of Nagata and Yamashita [91]. The sample is extracted with acetone and hexane (4:6), and the optical density of the supernatant is measured at 505 nm by spectrophotometer. The lycopene contents were calculated as mg/100 g FW.

### Ethanolic extract

In brief, 5.0 g of tomato samples were crushed by liquid nitrogen, and after that, 25 ml of 80% ethanol was added, and the mixture shaken for 24 h at 25 °C. After filtration, the extraction was carried out two times [92]. The obtained ethanolic extract volume was completed and utilized for the evaluation of free phenols, flavonoid, and antioxidant activity (DPPH).

### Total phenolic contents

The ethanolic extract of tomato was used to determine phenolic content according to the method described by Shahidi and Naczk [93]. One ml of ethanolic extract with 0.5 ml of Folin Ciocalteu reagent were well mixed in dry test tube thoroughly shaken for 3 min and 1.0 ml of saturated Na_2_CO_3_ solution was added and well mixed then 3.0 ml of distilled water were added. After one hour phenolic compounds were determined by reading the developed blue color at 725 nm. The results were represented as mg gallic acid/g FW.

### Total flavonoid content

The aluminum chloride colorimetric method was utilized to determine the total flavonoid content in ethanolic extract of tomato as described by Marinova et al. [94]. Each ethanolic extract (1.0 ml) or standard solution of quercetrin was added to 10 ml volumetric flask containing 4.0 ml distilled water. To the flask 0.3 ml of 5% NaNO_2_ was added. After 5 min, 0.3 ml of 10% AlCl_3_ was added and after 6 min, 2.0 ml 1 M NaOH was added and the total volume was made up to 10 ml with distilled H_2_O. The solution was mixed well and the absorbance was measured against the blank at 510 nm. and total flavonoids were expressed as mg quercetin/g FW.

### Antioxidant activity by DPPH radical

According to Gulluce et al. [95], the constant radical 2, 2′-diphenylpicrylhydrazyl (DPPH) is the reagent used in this spectrophotometric experiment and the absorbance was read against the blank at 517 nm. Results were expressed as inhibition percentage of the DPPH radical (antioxidant activity %).

### Water soluble vitamins by HPLC

Vitamins of cherry tomato samples were analyzed by Agilent 1260 infinity HPLC Series (Agilent, USA), equipped with Quaternary pump, a column: Kinetex XB-C18 100 mm x 4.6 mm (Phenomenex, USA), operated at 35 °C. The separation is accomplished by a binary linear elution gradient with (A) 25 mM NaH_2_PO_4_ pH = 2.5, and (B) methanol. The injection volume was 20 µl and the flow rate was 0.5 ml min^− 1^. The detection was achieved by a VWD detector set at 254 nm for vitamin C and 220 nm for vitamins B3, B6, B9, and B12. The water soluble vitamins were identified by comparison with authentic standard retention times [96], and the vitamin contents were expressed as mg/kg FW.

### Statistical analysis

The study was conducted using a randomized complete block design (RCBD) with three replicates. Data were reported as the mean of three measurements ± SD. The MSTAT-C software (Michigan State University, USA) was used to analyze the data using analysis of variance (ANOVA). Duncan’s multiple range tests [97] were then used to further distinguish between the means at *P* ≤ 0.05.

## Data Availability

The data sets used and/or analyzed during the current study available from the corresponding author on reasonable request.
